# Allelopathy in Durum Wheat Landraces as Affected by Genotype and Plant Part

**DOI:** 10.3390/plants11081021

**Published:** 2022-04-08

**Authors:** Aurelio Scavo, Gaetano Pandino, Alessia Restuccia, Paolo Caruso, Sara Lombardo, Giovanni Mauromicale

**Affiliations:** Department of Agriculture, Food and Environment (Di3A), University of Catania, 95123 Catania, Italy; aurelio.scavo@unict.it (A.S.); a.restuccia@unict.it (A.R.); pcamps@libero.it (P.C.); g.mauromicale@unict.it (G.M.)

**Keywords:** allelopathy, durum wheat, weed management, seed germination, polyphenols, flavonoids, *Portulaca oleracea*, *Stellaria media*

## Abstract

Durum wheat is one of the largest cultivated crops across Mediterranean areas. The high demand for sustainable crop productions, especially concerning weed management, is driving the return to local landraces. In the present work, the in vitro allelopathic effects of the extracts of three durum wheat landraces (‘Timilia’, ‘Russello’ and ‘Perciasacchi’) and a modern variety (‘Mongibello’), obtained from three different plant parts (ears, stems and roots), were tested on seed germination (G) and mean germination time (MGT) of *Portulaca oleracea* L. and *Stellaria. media* (L.) Vill., two weeds commonly infesting wheat fields. In addition, the total polyphenol (TPC) and total flavonoid (TFC) content of extracts was determined. All extracts reduced G and increased MGT in both weeds compared to the control. The magnitude of phytotoxicity was strongly affected by the influence of genotype, plant part and extract dilution. Overall, the landraces ‘Timilia’ and ‘Russello’ showed the highest allelopathic effects, ear extracts were the most active, and the maximum extract dilution induced higher phytotoxicity. Extracts’ TPC and TFC corroborated these results. The findings obtained here encourage the use of local landraces as a source of allelochemicals and suggest that they could be left on soil surface or soil-incorporated after harvest for a possible weed control.

## 1. Introduction

Wheat is the most important grain crop worldwide, native to South-East Asia and widely cultivated since prehistoric times in the temperate zones. Nowadays, the world harvested area is about 215 × 10^6^ ha with ~765 × 10^6^ Mg of grain [1]. Most of these data refer to the hexaploidy species *Triticum aestivum* L. (bread wheat), while the only tetraploid (2n = 4x = 28) species of economic importance is durum wheat [*Triticum turgidum* subsp. *durum* (Desf.) Husn.]. It is mainly grown in the European Union (EU) on above 2.1 × 10^6^ ha with a 7.6 × 10^6^ Mg grain production, of which Italy is the main EU producer with 1.2 × 10^6^ ha and 3.8 × 10^6^ Mg harvested production [2]. Durum wheat is cultivated across the Mediterranean Basin and other semiarid regions, where it is appreciated for its high cooking quality and for the production of pasta, semolina, couscous, flatbread and bulgur [3,4]. In this area, local landraces are specifically adapted to environmental conditions and soil properties, so much so that the pool of Mediterranean landraces contains the largest genetic diversity within the species [5]. These landraces were largely cultivated for centuries, when in the middle of the 20th century they were progressively replaced with more productive and genetically-improved semi-dwarf cultivars. In addition to this, durum wheat yields have been consistently enhanced thanks to the advancements in the agronomic management, in particular herbicide application. Despite some differences between durum and bread wheat in the response to herbicides, both species need considerable amounts of herbicides against monocotyledonous and dicotyledonous weeds [3,6]. However, the irrational use of herbicides caused the development of resistance and shifts in weed populations, the emergence of a substitution weed flora, an important environmental pollution and subsequent health hazards [7]. Exploring the application of alternative and sustainable strategies for weed management in wheat agroecosystems has therefore become mandatory. Within this scenario, allelopathy is a novel tool that is gaining more and more popularity across the scientific community [8].

Allelopathy, a term firstly coined by the Austrian physiologist Hans Molisch in 1937 to indicate the biochemical interactions between all plants, refers to any direct or indirect, beneficial or detrimental effect by one plant on another through the release of chemical compounds into the environment [9]. These chemical compounds, known as allelochemicals, are secondary metabolites or waste products of primary metabolic pathways produced by living organisms, including plants and microorganisms, belonging to different chemical classes and playing a defensive role for the plant [10]. There are different mechanisms by which allelopathy can be exploited to manage weeds: crop rotation with allelopathic species, cover cropping, green manuring, intercropping and use of plant extracts [11]. The latter, in particular, have been largely adopted alone or in combination with reduced doses of herbicides. Increase of reactive oxygen species (ROS) production, alteration of cell structure and membrane permeability, alteration of photosynthesis and respiration, reduction and/or inhibition of seed germination and seedling growth, are extensively documented as common effects of plant extract [12].

Allelopathy in wheat has been deeply studied and demonstrated by a consolidated scientific literature [13,14]. There are many allelochemicals involved in wheat allelopathy, belonging to three main chemical classes: phenolic acids (e.g., *p*-hydroxybenzoic, ferulic, syringic, vanillic, *p*-coumaric, etc.), benzoxazinoids (DIMBOA, MBOA, HMBOA, DIMBOA glycoside, BOA) and short-chain fatty acids (e.g., propionic, acetic, butyric, etc.) [13,14]. The allelopathic traits and the synthesis of these allelochemicals are genetically controlled and characterised by a high polygeneticity. For instance, it is known that the genes coding for benzoxazinoids (DIMBOA) accumulation are located on chromosomes 4A, 4B, 4D and 5B [15]. In addition, Wu et al. [16] identified two major quantitative trait loci on chromosome 2B related to wheat allelopathy. Allelopathic genetic variability among wheat cultivars is very common and the breeding of cultivars with improved allelopathic potential is now under investigation [17]. One of the first studies is that of Spruell [18], who screened 286 bread wheat accessions for their allelopathic effects against *Bromus japonicus* L. and *Chenopodium album* L. Later, Wu et al. [19] firstly evaluated the allelopathic potential of 92 wheat cultivars against annual ryegrass, and then screened 453 wheat accessions from 50 countries, reporting a 10 to 91% range of root growth inhibition [20]. The production and amount of allelochemicals as expression of the allelopathic potential varied in relation to plant parts and plant age. For instance, it was found that the concentration of benzoxazinoids in wheat seeds was similar to that in foliage and roots [21], whereas Mogensen et al. [22] reported a lower concentration of DIMBOA in roots than in leaves. Generally, the concentrations of these compounds decline with plant age [22]. The allelopathic effects of wheat extracts were investigated in laboratory by testing the effects of aqueous and straw extracts on seed germination and seedling growth of selected weeds, as well as under field conditions by evaluating its inclusion in crop sequences and residue incorporation [13,14]. However, most of these studies refer to bread wheat straw and other plant parts, while durum wheat allelopathy has been very little studied.

Given the well-known effect of genotype and plant part on the allelopathic expression of plants, the return to local landraces by virtue of their genetic importance and market demand, and considering also the higher weed suppressive ability of landraces than modern cultivars, we hypothesise that durum wheat landraces could be more allelopathic than modern cultivars, with genotype- and plant part-dependent allelopathic effects. Indeed, in our experience, fields of landraces show a lower weed density than modern ones. To test these hypotheses, a systematic study was performed with the aim of screening the allelopathic potential of three durum Sicilian wheat landraces compared to a modern variety by testing the allelopathic effects of root, stem and ear extracts on two weed species commonly infesting wheat fields (*Portulaca oleracea* L. and *Stellaria media* (L.) Vill.). Furthermore, the total polyphenol content (TPC) and total flavonoid content (TFC) of durum wheat extracts were determinate to detect possible interactions between extracts phytotoxicity and polyphenols amount.

## 2. Results

### 2.1. Allelopathic Effects of Durum Wheat Extracts

From the ANOVA, it emerged that the two target weeds were differently affected by durum wheat extracts (Table 1).

#### 2.1.1. Allelopathic Effects on *Portulaca oleracea*

The three-way interaction ‘wheat genotype × plant part × extract dilution’ was significant for both G (*p* ≤ 0.01) and MGT (*p* ≤ 0.05), with the ‘extract dilution’ showing the highest contribution to variance (58% and 57%, respectively) (Table 1).

**Figure 1:**
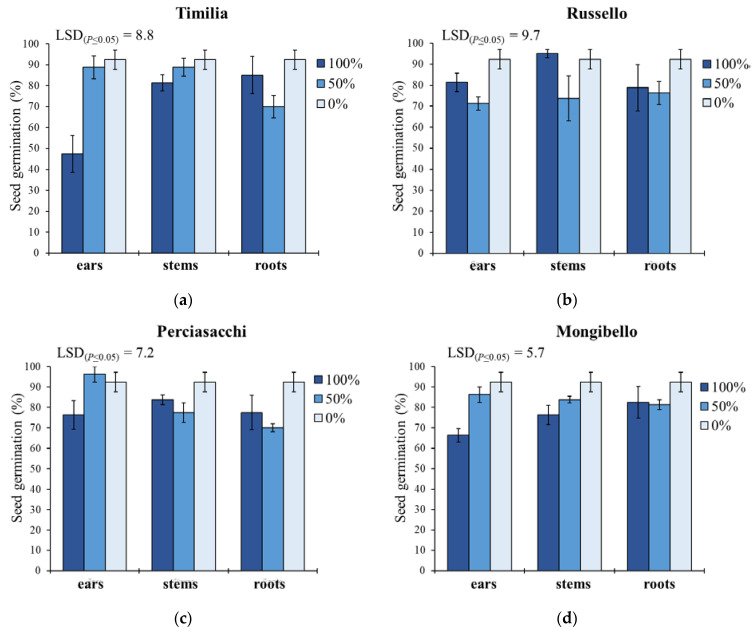
Seed germination percentage of *Portulaca oleracea* as affected by durum wheat genotype, plant part and extract dilution. (**a**) ‘Timilia’; (**b**) ‘Russello’; (**c**) ‘Perciasacchi’; (**d**) ‘Mongibello’; Least Significant Difference (LSD) interaction was calculated with the LSD test at *p* < 0.05. Bars indicate ± standard error.

Concerning G (Figure 1), ear extracts caused lower G values than stem and root extracts in ‘Timilia’ (respectively −12.9% and −7.6%) and ‘Russello’ (−6.2% and −1.0%), while in ‘Perciasacchi’ the roots extracts were the most allelopathic (80% vs. 88% of ears and 85% of stems). The lowest seed germination was observed with the 100% ear extract from ‘Timilia’ (47.5%), followed by the 100% ear extract from ‘Mongibello’ (66.3%); on the contrary, in ‘Russello’ the most active extract was that obtained from the ears diluted at 50% (71.3%), while in ‘Perciasacchi’, it was the 50% root extract (70%). Pooling over wheat genotypes and plant parts, pure extracts (100%) showed a higher inhibitory activity (77.5%) than 50% dilution (80.3%) and the control (92.4%).

**Figure 2:**
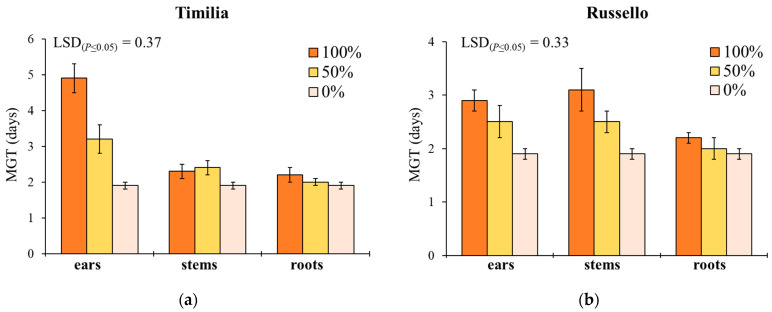

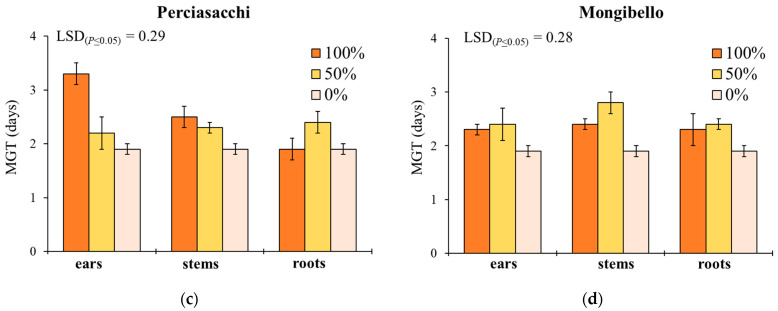
Mean germination time (MGT, days) of *Portulaca oleracea* as affected by durum wheat genotype, plant part and extract dilution. (**a**) ‘Timilia’; (**b**) ‘Russello’; (**c**) ‘Perciasacchi’; (**d**) ‘Mongibello’; Least Significant Difference (LSD) interaction was calculated with the LSD test at *p* < 0.05. Bars indicate ± standard error.

Mean germination time showed a similar response to G, with the exception of the significant effect provided by plant part (Table 1). In ‘Timilia’ and ‘Perciasacchi’, the ear extracts at 100% caused the highest MGT (4.9 and 3.3 days, respectively), whereas the 100% and 50% stem extracts were, respectively, the most active in ‘Russello’ (3.1 days) and ‘Mongibello’ (2.8 days) (Figure 2). Regardless of wheat genotypes and extract dilution, the extracts obtained from ears determined a higher MGT than stems and roots (2.5 vs. 2.2 and 2.1 days, respectively). Moreover, 100% dilution increased more MGT than 50% and 0% (2.7 vs. 2.3 and 1.9 days, respectively).

#### 2.1.2. Allelopathic Effects on *Stellaria media*

**Figure 3:**
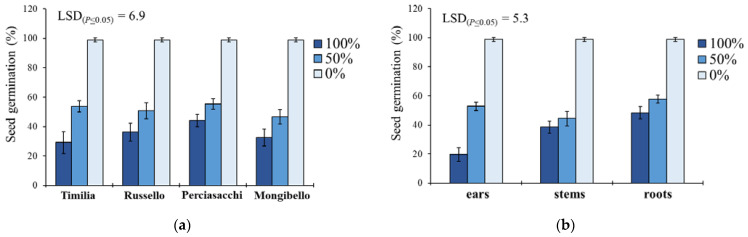
Effect of durum wheat ‘genotype × extract dilution’ (**a**) and ‘plant part × extract dilution’ (**b**) interactions on *Stellaria media* seed germination percentage. Least Significant Difference (LSD) interaction was calculated with the LSD test at *p* < 0.05. Bars indicate ± standard error.

In *S. media*, seed germination was significantly affected by the two-way ‘wheat genotype × extract dilution’ and ‘plant part × extract dilution’ interactions (Table 1). Regarding the former (Figure 3a), the trend 0% < 50% < 100% was constant for all the wheat genotypes, with an average reduction of 64% (pure extracts) and 48% (extracts diluted at 50%) of G compared to the control. ‘Timilia’ extracts at 100% showed the highest allelopathic effect (29.2% vs. 37.5% on the average of the other three genotypes), while ‘Perciasacchi’ extracts at 50% had the lowest (55.3%). Concerning the ‘plant part × extract dilution’ interaction, a trend of 0% < 50% < 100% was found for the three plant parts (Figure 3b). Among the maximum dilutions (100%), ear extracts caused the lowest G (19.7%), whereas stem extracts were the most efficient in terms of G reduction among the 50% dilutions (44.4%). Root extracts showed the lowest inhibitory activity at both 100% and 50% dilution.

**Figure 4:**
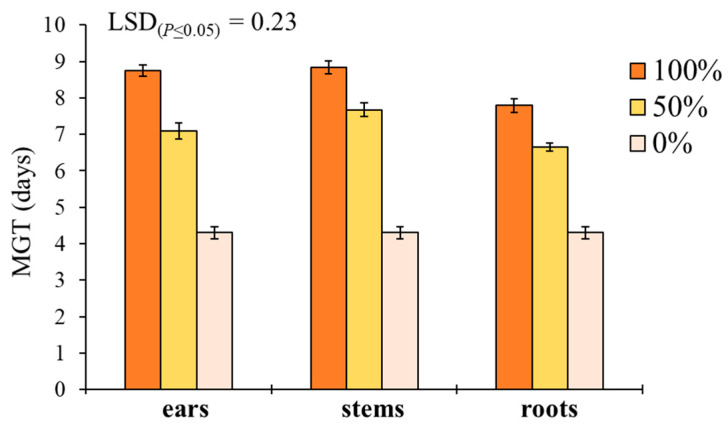
Effect of durum wheat ‘plant part × extract dilution’ interaction on *Stellaria media* mean germination time (MGT, days). Least Significant Difference (LSD) interaction was calculated with the LSD test at *p* < 0.05. Bars indicate ± standard error.

‘Plant part × extract dilution’ was the only significant interaction affecting MGT in *S. media* (Table 1). Pure extracts at 100% better performed than 50% and 0% dilutions in increasing the MGT for both ears, stems and roots (Figure 4). Stem extracts determined the highest MGT at both 100% (8.8 days) and 50% (7.7 days) dilution, followed by ears (8.6 and 7.1 days) and roots (7.8 and 6.6 days).

#### 2.1.3. Synthesis of the Allelopathic Effects

Table 2 shows the allelopathic effect response index (RI) and the synthesis effect (SE) of main factors for the two target weeds. RI values are not described since they are still incorporated in SEs. The synthesis effect of wheat genotype was significant for both weeds. In particular, ‘Timilia’ showed a significantly higher SE than the other genotypes in *P. oleracea*. Similarly, significantly higher SEs were obtained by ‘Timilia’ and ‘Mongibello’ extracts in *S. media*. ‘Perciasacchi’ extracts showed the lowest SEs in both weeds. Regarding plant part, SE of ears was significantly higher than stems and roots in *P. oleracea*; also, *S. media* roots showed the significantly lowest SE. Moreover, it can be easily seen that the allelopathic effect was enhanced by increasing the dilution of extracts (0.45 vs. 0.36 SE in *P. oleracea* and 1.13 vs. 0.87 SE in *S. media*).

### 2.2. Total Polyphenol (TPC) and Flavonoid Content (TFC) of Durum Wheat Extracts

**Figure 5:**
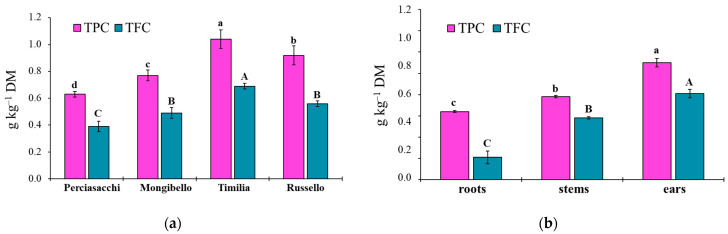
Total polyphenol (TPC) and total flavonoid (TFC) content in roots, stem and ears of durum wheat extracts in relation to the genotype (**a**) and plant part (**b**). Different letters (a–d) indicate significant differences for the TPC (*p* ≤ 0.05). Different letters (A–C) indicate significant differences for the TFC (*p* ≤ 0.05). Bars indicate ± standard error. DM: dry matter.

**Figure 6:**
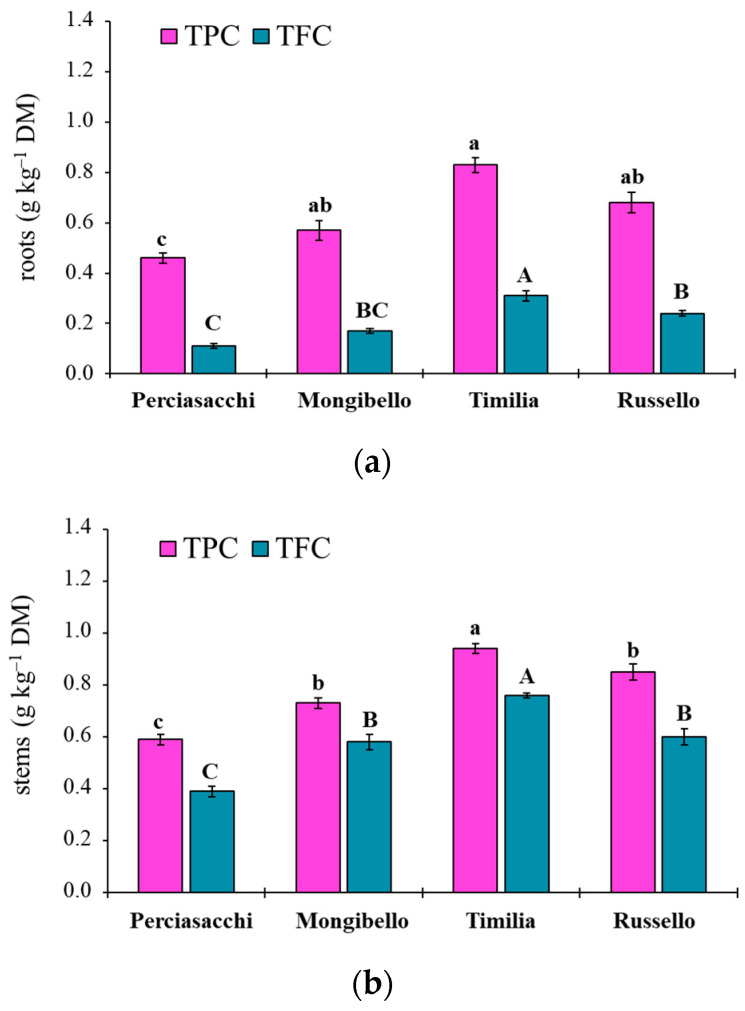

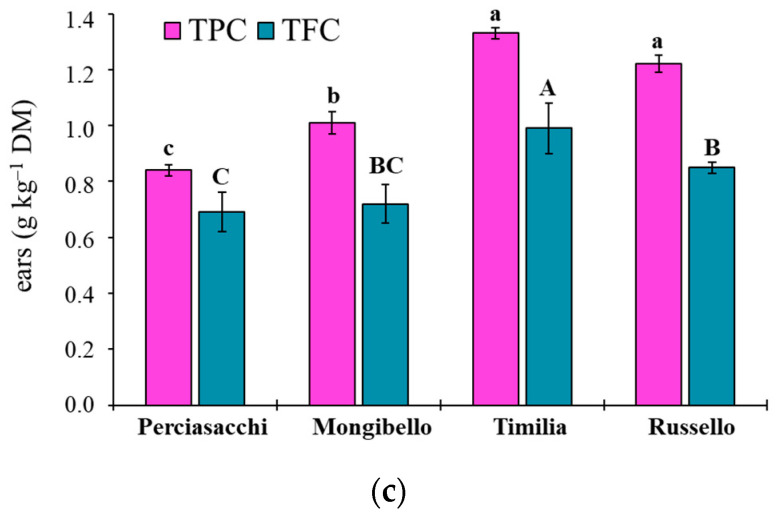
Total polyphenol (TPC) and total flavonoid (TFC) content in root (**a**), stem (**b**) and ear (**c**) extracts of durum wheat genotypes. Different letters (a–c) indicate significant differences among genotypes for the TPC (*p* ≤ 0.05). Different letters (A–C) indicate significant differences among genotypes for the TFC (*p* ≤ 0.05). Bars indicate ± standard error. DM: dry matter.

The ANOVA showed that both TPC and TFC values of durum wheat extracts were significantly affected by both durum wheat genotypes (*p* < 0.0001 TPC, TFC) and plant parts (*p* < 0.0001 TPC, TFC), whereas any two-way interaction was significant (*p* = 0.1584 and *p* = 0.3459, respectively). Regardless of plant part, ‘Timilia’ showed the highest TPC and TFC values (1.04 and 0.69 g kg^−1^ DM, respectively), followed by ‘Russello’, ‘Mongibello’ and ‘Perciasacchi’ (Figure 5a). In relation to the plant part, the trend of ears > stems > roots was found for both TPC and TFC, with ear extracts showing a +72% of TPC and +286% of TFC compared to root extracts (Figure 5b). In detail, ‘Timilia’ showed the highest TPC values in all plant parts (1.33 g kg^−1^ DM in ears, 0.94 g kg^−1^ DM in stems and 0.83 g kg^−1^ DM in roots), followed in decreasing order by ‘Russello’ (1.22, 0.85 and 0.68 g kg^−1^ DW, respectively, in ears, stems and roots), ‘Mongibello’ (1.01, 0.73 and 0.57 g kg^−1^ DM) and ‘Perciasacchi’ (0.84, 0.59 and 0.46 g kg^−1^ DM) (Figure 6). The same trend was found for TFC, with ‘Timilia’ and ‘Perciasacchi’ showing, respectively, the highest and the lowest values (Figure 6).

## 3. Discussion

Most of the research about wheat allelopathy have been conducted on bread wheat and on the allelopathic effects of wheat crop residues, leachates and mulch/cover crop [13,14]. Durum wheat allelopathy, on the contrary, is still poorly understood. This study demonstrated that durum wheat extracts have a significant allelopathic activity against two common weeds infesting wheat fields. The allelopathic activity was evaluated in terms of weed G and MGT, which are two well-known secondary expressions derived from primary effects such as the increase of reactive oxygen species (ROS), reduction or inactivation of the physiological activity of phytohormones, alteration of cell membrane permeability, division and elongation [12]. In particular, plant extracts generally inhibit seed germination by disrupting mitochondrial respiration and oxidative pentose phosphate pathways [23]. The phytotoxicity level varied in relation to genotype, plant part and extract dilution, as commonly found in many other similar studies. Genotype- and dose-response allelopathic effects are widely reported in the literature. Scavo et al. [24,25], for instance, indicated that the allelopathic activity of *Cynara cardunculus* L. depends on the botanical variety—with the wild cardoon being more phytotoxic than cultivated cardoon and globe artichoke—and extract concentration. Allelopathic genetic variability has been thoroughly studied in bread wheat [18,19,20], while to the best of our knowledge this is the first time in durum wheat. The allelopathic activity of two Tunisian durum wheat varieties, ‘Karim’ and ‘Om rabii’, was evaluated by Oueslati [26] on seed germination and seedling growth of barley and bread wheat. The same author, investigating the effect of plant part, also found that leaf extracts were the most active, whereas root and stems extracts showed no effect in reducing radicle length and seed germination.

Here, two local landraces (‘Timilia’ and ‘Russello’) showed higher allelopathic effects than a modern variety (‘Mongibello’) on both target weeds. Moreover, ear extracts provided better results in terms of phytotoxicity than stems and roots. These findings were corroborated by the phytochemical analysis of aqueous extracts. Indeed, significantly higher TPC and TFC values were detected in ‘Timilia’ and ‘Russello’ extracts. Moreover, both TPC and TFC followed the trend ears > stems > roots, as found for phytotoxic effects. Wheat polyphenols mainly include hydroxybenzoic and hydroxycinnamic acid derivatives such as *p*-hydroxybenzoic, ferulic, syringic, vanillic, caffeic and *p*-coumaric [4,27]. They were detected in the whole grains and in bran fractions, while no information was available for other wheat plant parts. In general, these secondary metabolites are produced as a defence mechanism in adaptation to biotic and abiotic stresses (water-deficit and high intensity of solar radiation in Mediterranean environments). In contrast with Bertholdsson [28], who underlined how bread wheat landraces and old cultivars were less allelopathic than modern varieties, in this study the landraces ‘Timilia’ and ‘Russello’ were more allelopathic than the modern variety ‘Mongibello’. This is not strange since agronomic practices such as herbicide application have resulted in the competitive loss of modern cultivars with weeds. Giambalvo et al. [29], in fact, suggested the choice of old and tall landraces such as ‘Russello’ in weedy and low-N conditions due to their high competitive capacity with weeds. The higher weed-suppressive ability of old landraces compared to modern varieties could be therefore attributable not only to competition, but also to allelopathy, as demonstrated in this study. The decrease of G and increase of MGT mediated by allelochemicals is a strategy adopted by certain crops, such as durum wheat landraces, to win the competition with weeds, thus decreasing the herbicides supply.

## 4. Materials and Methods

### 4.1. Location and Agronomic Management of Wheat Field

Durum wheat genotypes were cultivated in the experimental farm of the University of Catania, located in Eastern Sicily (South Italy, 37°25′ N; 15°30′118″ E; 10 m a.s.l.). The soil, Typic and/or Vertic Xerochrepts (Soil Survey Staff, 1999), showed the following physio-chemical properties in the 0–40 cm profile: sand 27%, clay 45%, silt 28% (clay texture), organic matter 1%, total N 1.1 g kg^−1^, available P_2_O_5_ 10 mg kg^−1^, exchangeable K_2_O 300 mg kg^−1^, pH 8.1 and cation exchange capacity 169 meq 100 g^−1^. The climate of the zone is typically semiarid Mediterranean, characterised by ~500 mm of annual precipitations, mild rain winters and hot dry summers.

Durum wheats under study included three Sicilian landraces (‘Timilia’, ‘Russello’ and ‘Perciasacchi’) and a modern variety (‘Mongibello’), recently bred by the University of Catania from a ‘Trinakria × Valforte’ cross. They are autumn-sowing genotypes with late or medium-late maturity, and mean yields in Sicily ranged between 1300–2800 kg ha^−1^. Plants were arranged in a randomized block design with three replicates, within a 35 × 36.5 m experimental area. Each cv. had a net plot size of 30 m^2^, for a total of 12 plots of 10 m^2^ with 6 rows spaced 21 cm apart. Sowing was carried out in December 2018 by means of a self-propelled plot seeder (Winterstaiger, Ried, Austria) at the rate of 400 viable seeds m^−2^ to reach a mean target ear density of ~300 ears m^−2^.

Wheat genotypes were grown by applying a low-input agricultural management. Seedbed preparation was realized with a shallow hoeing (20 cm deep) in early autumn followed by a disk harrow. The fertilization program consisted of 54 kg N ha^−1^ and 108 kg P_2_O_5_ ha^−1^ before sowing, combined with 26 kg N ha^−1^ (ammonium nitrate, 27% N) at tillering stage. Weeds were controlled by hand and by brush cutter.

### 4.2. Sampling of Plant Materials and Extracts Preparation

About 30 plants for each variety were randomly sampled in June 2019 at full maturity stage. In the laboratory, the plants were washed with tap water and separated into roots, stems and ears. Extracts were prepared by combining the methodologies proposed by Oueslati [26] and Wu et al. [20], with some modifications. In detail, the three plant parts were finely chopped and dried in an oven at 45 °C up to constant weight. Ten grams of DM of each plant material were soaked with 150 mL of distilled water for 48 h at room temperature (20 °C ± 1) in the dark. Then, the mixtures were filtered through a Whatman no. 2 filter paper and centrifuged at 200 rpm for 15 min at 10 °C to remove debris. Finally, each extract was diluted with distilled water to obtain three final dilutions: 100% (pure extracts), 50% and 0% (distilled water) as control. The prepared extracts were stored in a refrigerator at 3 °C for further uses.

### 4.3. Seed Collection and Germination Bioassay

The allelopathic effects of the above-mentioned durum wheat extracts were tested on seed germination of *P. oleracea* and *S. media*. The former is a cosmopolitan spring–summer annual weed, therophyte, belonging to the Portulacaceae family; the latter is a Caryophyllaceae member, biennial and hemicryptophyte weed, with an autumn–winter cycle. Both weeds highly infest Mediterranean durum wheat fields, where they exert a severe pressure, respectively, at the end and at the beginning of the crop’s biological cycle. The seeds of *P. oleracea* were collected around the experimental farm of the University of Catania, whereas *S. media* seeds derived from natural populations sited in Calatabiano (Sicily, 37°49′ N, 15°13′ E; 50 m a.s.l.). Mature collected seeds were cleaned from inert materials (debris, pebbles, etc.), selected for size and colour homogeneity with a MS5 Leica stereomicroscope (Leica Microsystems, Wetzlar, Germany), and stored in paper bags at room temperature.

Germination bioassays were carried out in 9 cm Petri dishes by moistening a double Whatman no. 2 layer with 5 mL of root, stem and ear extracts at two different dilutions (100% and 50%). Distilled water was used as a control. There were four replicates of 25 seeds for each extract and dilution. Petri dishes of *P. oleracea* were incubated in continuous darkness at 35 °C and wrapped with an aluminium foil, while *S. media* ones were kept in alternating light (dark/light cycle 14/10 h) at 17 °C. These temperatures and photoperiods are the optimal conditions for seed germination of each species [24,30]. In both cases, Petri dishes were sealed with parafilm to prevent evaporation of the solution. Seed germination was counted daily, considering the seeds as germinated when the radicle protruded over 2 mm. Germination bioassays ended when no seeds had germinated for 3 consecutive days.

### 4.4. Total Polyphenol Content of Aqueous Extracts

The total polyphenol content was quantified using a modified method proposed by Pandino et al. [31]. About 10 mL of each extract was evaporated under vacuum using a rotary evaporator (Buchi rotavapor). The residue was redissolved in 1.5 mL methanol 80% and stirred at room temperature for 1 h, with shaking. The mixture was centrifuged at 5000× *g* for 5 min at 25 °C. A diluted aliquot was mixed with Folin—Ciocalteu reagent at room temperature for 2 min. Sodium carbonate (5%, *w*/*v*) was added and the mixture was allowed to rest at 40 °C for 20 min in thermostatic bath. The absorbance was read at 725 nm by a Shimadzu 1601 UV–Visible spectrophotometer (Shimadzu Corp., Tokyo, Japan). The content was determined on the basis of a standard calibration curve generated with known concentrations of ferulic acid. All data presented are mean values of two independent experiments and expressed as g kg^−1^ of DM.

### 4.5. Total Flavonoid Content of Aqueous Extracts

Flavonoid content of the extracts was quantified using the aluminium chloride assay method performed by Zendehbad et al. [32]. In brief, 500 μL of redissolved extract was dissolved in 1.5 mL of ethanol (95%) and 0.1 mL of 10% aluminium chloride. Then, 0.1 mL of 1 M sodium acetate were added. The volume was made up to 5 mL with bidistilled water. The absorbance was measured at 725 nm by a spectrophotometer at 415 nm after 30 min. The content was determined on the basis of a standard calibration curve generated with known concentrations of rutin. All data presented are mean values of two independent experiments and expressed as g kg^−1^ of DM.

### 4.6. Data Analysis

In order to evaluate the allelopathic effects of wheat extracts on seed germination of the two target weeds, the following parameters and indices were considered:

(1) $$G\;\left(\%\right)=\left(\frac{n_{i}}{N}\right)\times 100$$

(2) $$\mathrm{MGT}\;\left(\mathrm{days}\right)=\left(\frac{\sum_{i=1}^{k}n_{i}t_{i}}{\sum_{i=1}^{k}n_{i}}\right)$$

(3) $$\mathrm{RI}=\left[\left(\frac{T}{C}\right)-1\right]\mathrm{if}\;T<C\;\;\;or\;\;\;\mathrm{RI}=\left[1-\left(\frac{C}{T}\right)\right]\mathrm{if}\;T\geq C$$

(4) $$\mathrm{SE}=RI_{\left|G\right|}+RI_{\left|MGT\right|}$$

where: G = final germination percentage; n_i_ = number of seeds germinated in the ith time; N = total number of seeds used in each Petri dish; MGT = mean germination time; t_i_ = time from the start of the experiment to the ith observation; RI = allelopathic effect response index; T = treatment value; C = control value; SE = allelopathic synthesis effect. Equation (2) was computed according to Ranal et al. (2009). RI was determined following Williamson and Richardson (1998), with positive values indicating stimulation by treatments and negative values indicating inhibition. SE, which represents the intensity of the allelopathic effect, was calculated as the sum of the corresponding absolute value of RI for germination percentage (|G|) and mean germination time (|MGT|), in accordance with Ma et al. (2020). Laboratory experiments, repeated twice, were arranged in a completely randomized design (CRD) with four replications.

### 4.7. Statistical Analysis

Data were analysed using analysis of variance (ANOVA) followed by the Fisher’s protected LSD test for means multiple comparisons. Statistically significant differences were set at *p* ≤ 0.05. Deviations from normality and homoscedasticity were determined before ANOVA, respectively, by graphically inspecting the residuals and with the Bartlett’s test. In particular, to meet the basic assumptions for linear models, G and MGT data were log_(x+1)_-transformed in accordance with Scavo et al. (2020). Mean ± standard error of untransformed data is presented and discussed. A generalized linear model (GLM) was initially applied considering ‘wheat genotype’, ‘plant part’, ‘extract dilution’ and ‘target weeds’ as main factors. Considering that the latter factor showed a high significance (*p* ≤ 0.001) for all the variables, data were therefore processed according to a generalized linear mixed model (GLMM) with ‘target weeds’ as a random factor. Two-way ANOVAs were conducted for the second order interactions between main factors, whereas one-way ANOVAs were applied to pooled RI and SE data. Data about TPC and TFC were analysed according to a factorial two-way ANOVA model (4 wheat genotypes × 3 plant parts). The CoStat^®^ software version 6.003 (CoHort Software, Monterey, CA, USA) was used for statistical analysis.

## 5. Conclusions

The present research documented the allelopathic effects of selected durum wheat landraces on seed germination of two common weed species infesting wheat (*P. oleracea* and *S. media*). The magnitude of phytotoxicity was related to genotype, plant part and extract dilution. In detail, two landraces (‘Timilia’ and ‘Russello’) were more allelopathic than the modern variety ‘Mongibello’, ears were more active than stems and roots, and seed germination was increasingly inhibited with increasing extract concentration. These findings were supported by extracts’ TPC and TFC, since their highest values were found in ‘Timilia’ and ‘Russello’ among genotypes, and ear extracts among plant parts. These results suggested that plant residues of local landraces could be left on the soil surface or soil-incorporated after wheat harvest for weed control. Furthermore, durum wheat landraces can be considered potential plants for the possible future production of bioherbicides. However, more research is required in this regard to identify, purify and isolate the allelochemicals involved in durum wheat allelopathy and to evaluate their effects under field conditions on a broader spectrum of weeds and soil seed banks.

## Figures and Tables

**Table 1:** *F*-Fisher values of main factors and their interactions resulting from analysis of variance (ANOVA) on final seed germination percentage (G) and mean germination time (MGT).

Source of Variation	Df	*Portulaca oleracea*		*Stellaria media*	
		G	MGT	G	MGT
*Main factors*					
Wheat genotype (G)	3	0.83 ns	1.45 ns	4.86 **	0.90 ns
Plant part (P)	2	2.74 ns	22.09 ***	28.81 ***	15.28 ***
Extract dilution (D)	2	28.39 ***	58.22 ***	292.44 ***	904.85 ***
*Interactions*					
(G) × (P)	6	2.21 *	4.50 ***	1.73 ns	1.93 ns
(G) × (D)	6	3.59 **	0.84 ns	3.18 **	1.66 ns
(P) × (D)	4	8.41 ***	11.80 ***	27.01 ***	4.75 **
(G) × (P) × (D)	12	2.83 **	2.28 *	0.79 ns	0.60 ns

Notes: *F*-Fisher values are referred to log_(x+1)_-transformed data; df: degrees of freedom; ***, ** and * indicate statistical significance at *p* ≤ 0.001, *p* ≤ 0.01 and *p* ≤ 0.05, respectively; ns: not significant.

**Table 2:** Allelopathic effect response index (RI) and synthesis effect (SE) of durum wheat extracts on final seed germination (G) and mean germination time (MGT) of *Portulaca oleracea* and *Stellaria media*. RI and SE values are pooled over main factors.

Main Factors		*Portulaca oleracea*			*Stellaria media*		
		RI		SE	RI		SE
		G	MGT		G	MGT	
**Wheat genotype**	*Timilia*	−0.17 a	0.25 a	0.46 a	−0.58 a	0.45 a	1.03 a
	*Russello*	−0.14 a	0.21 a	0.40 b	−0.56 a	0.43 a	0.99 a
	*Perciasacchi*	−0.12 a	0.18 a	0.39 b	−0.50 a	0.44 a	0.92 b
	*Mongibello*	−0.13 a	0.24 a	0.40 b	−0.60 a	0.43 a	1.02 a
**Plant part**	*ears*	−0.17 a	0.32 a	0.53 a	−0.63 a	0.46 a	1.08 a
	*stems*	−0.11 b	0.23 b	0.36 b	−0.57 a	0.47 a	1.04 a
	*roots*	−0.15 a	0.11 c	0.33 b	−0.45 b	0.40 b	0.86 b
**Extract dilution**	*100%*	−0.26 a	0.25 a	0.45 a	−0.64 a	0.49 a	1.13 a
	*50%*	−0.12 b	0.17 b	0.36 b	−0.48 b	0.38 b	0.87 b

Different letters between each column indicate statistical significance at *p* < 0.05 with the LSD test.

## Data Availability

All data are available via email request to the corresponding authors.
